# Screening and Identification for Immunological Active Components from Andrographis Herba Using Macrophage Biospecific Extraction Coupled with UPLC/Q-TOF-MS

**DOI:** 10.3390/molecules23051047

**Published:** 2018-04-30

**Authors:** Yaqi Wang, Jiaojiao Jiao, Yuanzhen Yang, Ming Yang, Qin Zheng

**Affiliations:** 1 Key Laboratory of Modern Preparation of Traditional Chinese Medicine, Ministry of Education, Jiangxi University of Traditional Chinese Medicine, Nanchang 330004, China; wangyaqi_3@163.com (Y.W.); yangyuanzhen666@163.com (Y.Y.); 2 College of Pharmacy, Chengdu University of Traditional Chinese Medicine, Chengdu 610072, China; jqiao6@163.com

**Keywords:** *Andrographis paniculata*, cell biospecific extraction, immunological activity, UPLC/Q-TOF-MS

## Abstract

The method of cell biospecific extraction coupled with UPLC/Q-TOF-MS has been developed as a tool for the screening and identification of potential immunological active components from Andrographis Herba (AH). In our study, a macrophage cell line (RAW264.7) was used to extract cell-combining compounds from the ethanol extract of AH. The cell binding system was then analyzed and identified by UPLC/Q-TOF-MS analysis. Finally, nine compounds, which could combine with macrophages, in an ethanol extract of AH were detected by comparing basic peak intensity (BPI) profiles of macrophages before and after treatment with AH. Then they were identified as Andrographidine E (**1**), Andrographidine D (**2**), Neoandrographolide (**3**), Dehydroandrographolide (**4**), 5, 7, 2′, 3′-tetramethoxyflavone (**5**), β-sitosterol (**7**), 5-hydroxy-7, 2′, 3′-trimethoxyflavone (**8**) and 5-hydroxy-7, 8, 2′, 3′-tetramethoxyflavone (**9**), which could classified into five flavonoids, three diterpene lactones, and one sterol. Their structures were recognized by their characteristic fragment ions and fragmentations pattern of diterpene lactones and flavonoids. Additionally, the activity of compounds **3**, **4**, and **7** was tested in vitro. Results showed that these three compounds could decrease the release of NO (*p* < 0.01) in macrophages remarkably. Moreover, **3**, **4**, and **7** showed satisfactory dose-effect relationships and their IC_50_ values were 9.03, 18.18, and 13.76 μg/mL, respectively. This study is the first reported work on the screening of immunological active components from AH. The potential immunological activity of flavonoids from AH has not been reported previously.

## 1. Introduction

Andrographis Herba (AH) refers to the Chinese herb Chuanxinlian, the dry aerial part of *Andrographis paniculata*, is widely used as a traditional medicine and health food in China, India, Thailand, and other southeast Asia countries for the treatment of dysentery, sore throat, and snakebites [1,2]. The diterpenes, especially andrographolide and dehydroandrographolide, were generally considered to be the main active constituents of AH. These molecules exhibited varying degrees of anti-inflammatory activities in vitro and in vivo. Clinically, andrographis injection exerts rapid and beneficial effects on upper respiratory tract infection, acute bacillary dysentery, and fever [3]. 

Nowadays, the use of AH for regulating immune response to treat severe sepsis, systemic lupus erythematosus, and other autoimmune diseases has gained increasing attention [4,5,6]. However, anaphylactoid reactions induced by andrographis injection have been reported with increasing frequency. In addition, recent studies have demonstrated that diterpenoid lactones, including andrographolide and dehydroandrographolide, could markedly increase the histamine level, cell degranulation rate, and release ratio of ammonia glycosidase, which may have a potential sensitizing capacity causing allergic reactions [3]. Therefore, it is supposed that AH may represent a bidirectional modulator in immune regulation. Moreover, flavones constitute the main chemical ingredients of AH. So far, nearly 70 flavones have been isolated from AH. Furthermore, some flavones have also revealed good inhibition effects on inflammation and thrombus [7,8]. Compared to the wide applications of diterpenes in the treatment of inflammation, the research on immunological active compounds is still very limited. Whether flavones also take part in immunological activity is not clearly known. Therefore, to better understand the immunomodulatory mechanism of AH, it is worthwhile to identify the other immunological active components in this herb. 

Modern pharmacological studies have indicated that the ability of drugs to interact with receptors or other targets on cell or membranes plays an important role in evaluating the effectiveness of drug absorption [9]. Therefore, the interactions of active molecules with target cells and cell-based affinity purification techniques have been employed as a screen for bioactive components in traditional Chinese medicine (TCM) [10,11,12]. Meanwhile, liquid chromatography coupled with time-of-flight mass spectrometry (LC-TOF-MS/MS) is a powerful and sensitive analytical technique for the identification of unknown molecules. In this paper, to screen potential immunological active components in AH, firstly macrophages, phagocyte cells that help initiate and are involved in all stages of immune responses, were used to extract cell-combining compounds from the ethanol extract of AH. Then the cell-binding system and structure elucidation of the cell-combining compounds were analyzed and identified by UPLC/Q-TOF-MS analysis. Finally, nine potential active candidate compounds were found to combine with macrophages. Their structures were recognized by comparing with reference standards and their MS/MS behavior. To verify the immunological activity, their effects on the cytokine release of macrophages were tested in vitro.

## 2. Results and Discussion

### 2.1. Macrophage Biospecific Extraction of Compounds in AH

In our study, macrophage RAW264.7 was used to extract cell-combining compounds in AH. 

**Figure 1:**
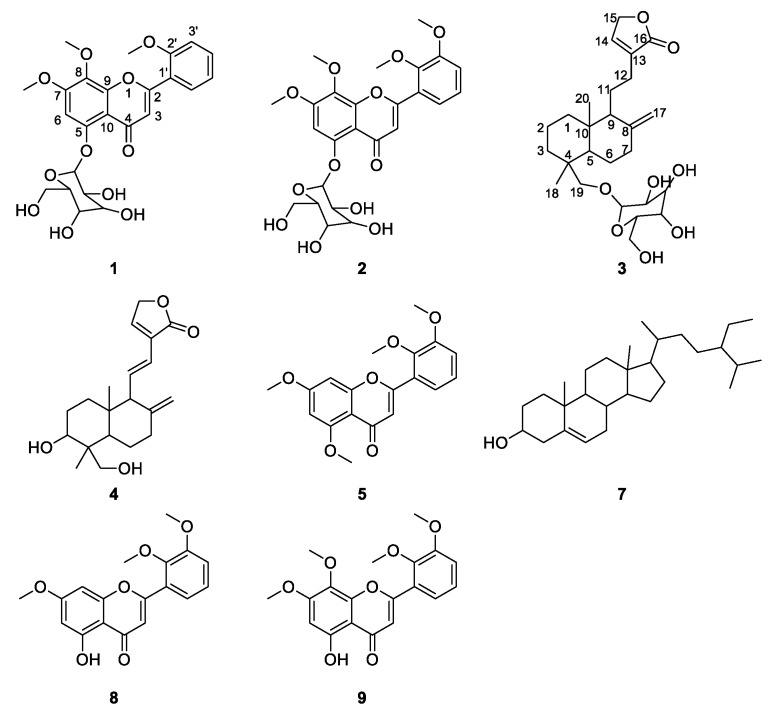
The structures of compounds identified in the cell extract of Andrographis Herba (AH). (**1**. Andrographidine E, **2**. Andrographidine D, **3**. Neoandrographolide, **4**. Dehydroandrographolide, **5**. 5,7,2′,3′-tetramethoxyflavone, **7**. β-sitosterol, **8**. 5-hydroxy-7,2′,3′-trimethoxyflavone, **9**. 5-hydroxy-7,8,2′,3′-tetramethoxyflavone).

**Figure 2:**
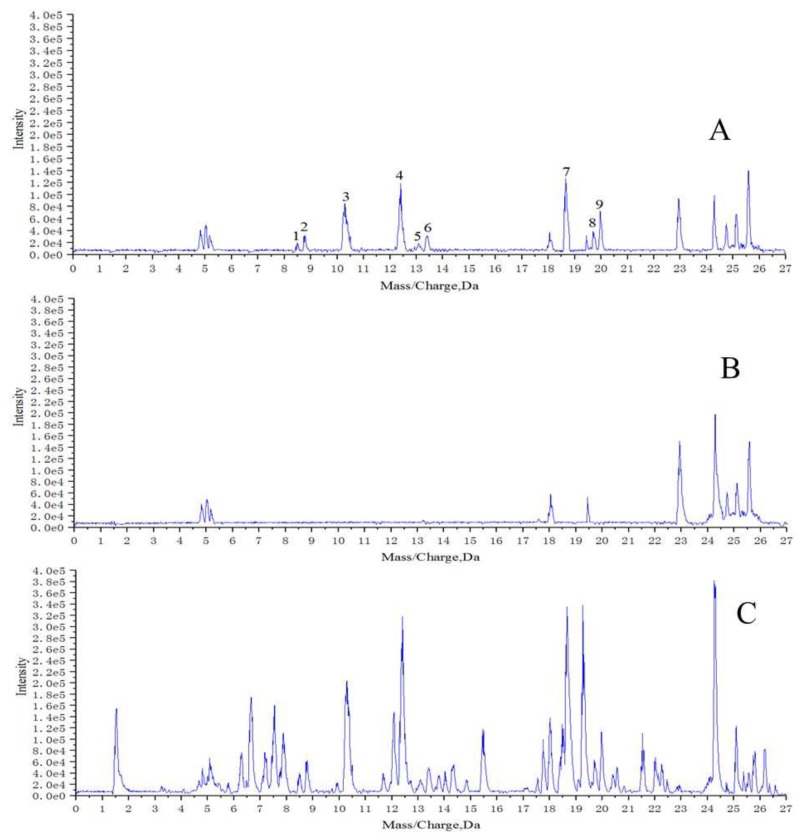
Base peak intensity (BPI) profiles of: (**A**) extract of AH treated with macrophages; (**B**) extract of denatured deposited macrophages; (**C**) ethanol extract of AH.

UPLC/Q-TOF-MS analysis was employed for the structure elucidation of the compounds extracted by macrophages (Figure 1). The base peak intensity (BPI) profiles of AH, the blank group, and the model group in positive ion mode are shown in Figure 2. It was found that the extract of denatured deposited RAW264.7 cells (blank group, Figure 2B) also contained complementary information at lower polar. This phenomenon may be caused by the existence of inorganic salts from PBS and DMEM. Thus, the extract of denatured deposited RAW264.7 cells was set as the blank group. Nine potential bioactive compounds were monitored in the model group (Figure 2A) by comparing MS/MS fragmentation behavior and retention time with AH (Figure 2C) and the blank group (Figure 2B). The results suggested that these nine components may have fine affinity to closely combine with some receptors or targets of macrophages, which could specifically interact with macrophages. When the macrophages were digested with absolute ethanol and were put under ultrasonic vibrations, the macrophages were ruptured and the cell-combining compounds were removed from the receptors or targets [9]. 

### 2.2. Identification of Active Components in AH

Eight compounds were identified according to reference standards and their MS/MS behaviors (Table 1). 

**Figure 3:**
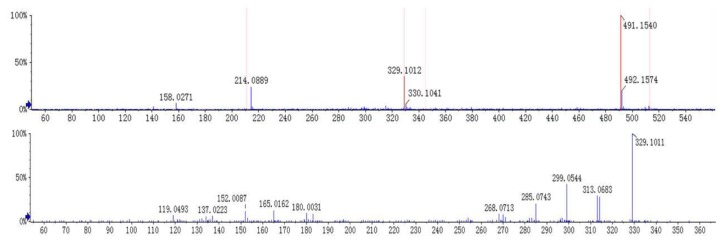
MS/MS spectra of andrographidine E (**1**).

**Figure 4:**
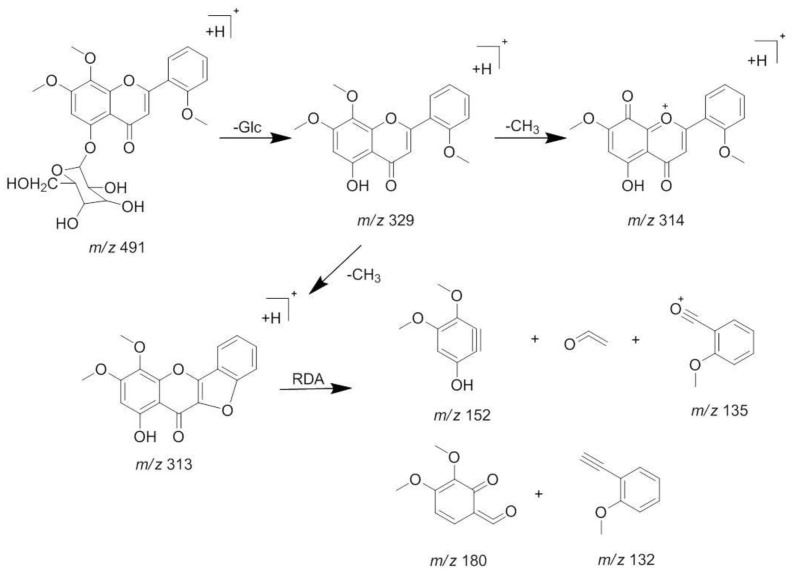
The proposed fragmentations pattern of andrographidine E (**1**).

The [M + H]^+^ at *m*/*z* 491 and [M + Na]^+^ at *m*/*z* 513 was deduced as a molecular ion of compound **1** (t_R_ 8.5 min). The MS^2^ spectrum of the ion at *m*/*z* 491 lost a characteristic ion 162 Da to generate [M + H − Glc]^+^ at *m*/*z* 329, which indicated that compound **1** may have the structure of glucose. Moreover, it was easy to lose 15 Da to generate the fragmentation of [M + H − Glc − CH_3_]^+^ at *m*/*z* 314 in its MS^3^ spectrum, indicating there is a methoxyl group at the C-6 or C-8 position. Besides, an ion at *m*/*z* 313 showed a similar abundance ratio in the MS^3^ spectrum, indicated there is a hydroxyl or methoxyl group at the C-2′ position. Based on the MS/MS behaviors of flavonoids, the fragmentations at *m*/*z* 183 in MS^3^ spectrum were generated by the Retro-Diels-Alder (RDA) fragmentation pattern. A series of losses of CO from the ketone group, C-fragmentation, and the loss of radicals such as CH_3_, CHO, and OH were also observed [13,14]. By comparing with the previous reports [15], compound **1** was identified as andrographidine E. The MS/MS spectra and the proposed fragmentation pattern of andrographidine E are shown in Figure 3 and Figure 4.

The fragmentation pattern of compound **2** was almost the same as that of **1**. Based on the MS spectra, it was apt to lose a characteristic ion 162 Da (Glc) to generate the aglycone at *m*/*z* 359. In the MS^2^ spectrum (Figure S1), the fragmentations of *m*/*z* 344 (loss of a CH_3_) and 329 (loss of two CH_3_) can be observed. In the MS^3^ spectrum, the fragmentations of *m*/*z* 315 (loss of a CO_2_) and 153 (RDA cleavage, loss of a C_10_H_10_O_2_ and CO_2_) were the same as the behavior of the MS/MS fragmentation pattern reported before [15]. By comparing the extract molecular weights with the chemical database, compound **2** was identified as andrographidine D.

**Figure 5:**
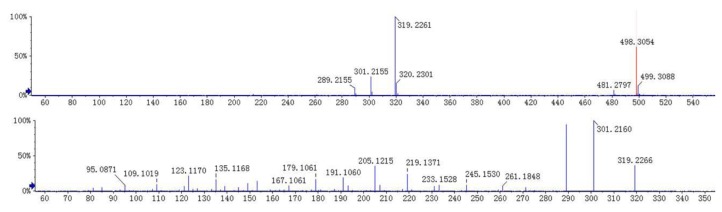
MS/MS spectra of neoandrographolide (**3**).

**Figure 6:**
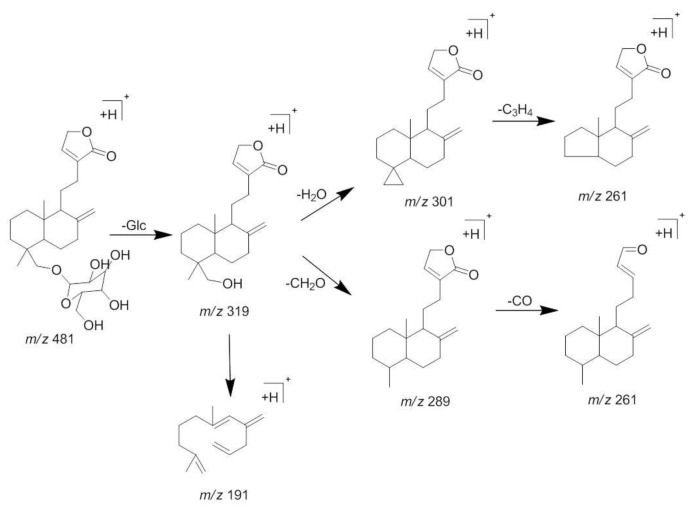
The proposed fragmentations pattern of neoandrographolide (**3**).

Compound **3** appeared at a retention time of 10.2 min, and ions at *m*/*z* 481 [M + H]^+^, 498 [M + NH_4_]^+^, 961 [2M + H]^+^, and 319 [M + H − Glc]^+^ were observed in its MS spectrum. Ions at *m*/*z* 319 [M + H − Glc]^+^ gave a typical fragment at *m*/*z* 301 [M + H − Glc − H_2_O]^+^ and 289 [M + H − Glc − CH_2_O]^+^ by losing an H_2_O molecule and a C-fragmentation in the MS^2^ spectrum. According to the MS/MS behaviors of diterpene lactones and by comparing with the reference standard, compound **3** could be characterized as neoandrographolide. The MS/MS spectra and the proposed fragmentation pattern of neoandrographolide are depicted in Figure 5 and Figure 6.

The [M + H]^+^ ion of compound **4** was detected at *m*/*z* 333.2058, indicating a molecular formula of C_20_H_28_O_4_ and the neutral loss of H_2_O from the hydroxyl group formed *m*/*z* 297 [M + H − 2H_2_O]^+^ (Figure S2). By comparing its MS behaviors with the reference standard, compound **4** could be recognized as dehydroandrographolide. 

Compound **5** was observed at 13.0 min. Ions at *m*/*z* 343, 328, 313, and 285 were observed, which were assigned as [M + H]^+^, [M + H − CH_3_]^+^, [M + H − 2CH_3_]^+^, and [M + H − 2CH_3_ − CO]^+^ ions, respectively. In the MS^2^ spectrum (Figure S3), characteristic ions at *m*/*z* 181 and 153, suggesting dimethoxy substitution, may be located in the flavone A ring. On the basis of the MS data from the literature [16], it was identified as 5, 7, 2′, 3′-tetramethoxyflavone.

The [M + H]^+^ at *m*/*z* 379 and [2M + H]^+^ at *m*/*z* 757 were deduced as molecular ions of compound **6**. The fragmentation pattern of compound **6** is similar to that of **4**. In the MS^2^ spectrum (Figure S4), molecular ion (*m*/*z* 379[M + H]^+^) yielded a predominant ion at *m*/*z* 343 by losing two units of H_2_O. Typical fragments at *m*/*z* 297, 285, 257, 175, 133 suggested that the compound **6** may be a diterpene derivative, similar to **4**. Unfortunately, this compound was not recognized despite our efforts.

Compound **7** shows a [M + H]^+^ ion at *m*/*z* 415, a [M + Na]^+^ ion at *m*/*z* 437 and a [2M + Na]^+^ ion at *m*/*z* 851, respectively. By comparing its MS behaviors (Figure S5) with the reference standard, compound **7** could be definitely identified as β-sitosterol.

Compounds **8** and **9** have similar fragmentation patterns to those of compounds **1** and **2**, respectively. In the MS^2^ spectrum (Figures S6 and S7), characteristic ions at *m*/*z* 314, 313, 299, 285, 180, 165 were produced both from compounds **8** and **9**. According to the MS/MS behaviors of flavonoids and the literature data [16], compounds **8** and **9** were identified as 5-hydroxy-7, 2′, 3′-trimethoxyflavone and 5-hydroxy-7, 8, 2′, 3′-tetramethoxyflavone.

### 2.3. Effects of Three Compounds on LPS-Induced Cytokine Release of Macrophages

Diterpene lactones and flavonoids are the main chemical components of AH. In this study, five flavonoids (**1**, **2**, **5**, **8**, and **9**), three diterpene lactones (**3**, **4**, and **6**) and a sterol (**7**) were extracted by macrophages. However, as their content in plants is low and difficult to extract, only compounds **3**, **4**, and **7** were extracted from AH. The effect of these three compounds on LPS-induced NO release of macrophages is shown in Table 2. Results indicated that the three compounds could decrease the release of NO in macrophages remarkably. Moreover, the three compounds showed satisfactory dose-effect relationships.

Although, there is no flavonoid isolate from AH, the flavonoids from AH have also been reported to regulate immune response and have attracted increasing attention as potential prophylactic drugs against a series of diseases, especially cancer and cardiovascular disease [17]. Moreover, compounds **8** and **9** have been reported to interact with several enzymes, such as detoxification enzymes and plasma proteins [17,18]. So, as the configuration isomers, compounds **1**, **2**, and **5** may also exhibit similar interaction with proteins. Thus, there may be a synergistic effect between flavonoids and diterpene lactones on the immune system.

## 3. Materials and Methods 

### 3.1. Chemicals and Reagents

AH was acquired from a local drugstore in Nanchang (China). Reference standards of andrographolide, dehydroandrographolide, neoandrographolide, and β-sitosterol (>98% purity, determined by HPLC) were purchased by the National Institute for the Control of Pharmaceuticals and Biological Products (Beijing, China). Ethanol (analytical grade), acetonitrile (chromatographic grade), and formic acid (chromatographic grade) were acquired from local chemical suppliers. Deionized water was prepared by a Milli Q-Plus system (Millipore, Bedford, MA, USA). Dulbecco’s modified Eagle medium (DMEM), lipopolysaccharide (LPS), and fetal bovine serum (FBS) were acquired from Gibco (Grand Island, NY, USA). 3-[4, 5-Dimethylthiazol-2-yl]-2, 5-diphenyl tetrazolium bromide (MTT) and DMSO were purchased by Beijing Solarbio Science and Technology Corporation (Beijing, China). Phosphate-buffered saline (PBS, pH7.4) was prepared in our laboratory. The murine macrophage cell line (RAW264.7 cells) was acquired from the Institute of Biochemistry and Cell Biology, Chinese Academy of Sciences (Shanghai, China).

### 3.2. Apparatus

An Agilent 6538 UPLC-ESI-Q-TOF-MS equipped with an online degasser, dual gradient pump, auto sampler, and column oven (Agilent Technologies, Palo Alto, CA, USA) was used for MS analysis. A Phenomenex reversed-phase Luna C_18_ column (150 × 2.0 mm, 3 μm) and a Phenomenex C_18_ guard column (Phenomenex, Torrance, CA, USA) were employed for chromatographic analysis. 

### 3.3. Preparation of AH Extracts

AH extracts were obtained according to the method described in the Chinese Pharmacopoeia. About 100 g dried AH was refluxing extracted with a 10-fold volume of 85% ethanol in water for 2 h, and this process was and repeated twice. After filtration, the extracts were evaporated to dryness at 45 °C under vacuum. Residues were dissolved in 100 mL 85% ethanol in water and filtered through a 0.22-μm membrane. The filtrate was then used as a sample for LC-MS analysis, and diluted with DMEM when treated with cells.

### 3.4. Cell Culture and Binding

RAW264.7 macrophages were cultured in DMEM supplemented with 10% FBS and antibiotics (100 U/mL penicillin and 100 g/mL streptomycin), and maintained at 37 °C in a humidified incubator containing 5% CO_2_. The medium was changed every two days until the cells reached confluence. Cell suspensions (1 mL, 1 × 10^6^ cells) were treated with the extracts of AH (1.90 mg/mL) for 6 h at 37 °C. Then the cells were washed four times with 2 mL PBS each time to scour off the possible non-selectively combining components. The eluates were discarded except for the last one, which was collected as a contrast for LC-MS analysis. Finally, the cells were digested by the addition of 1 mL absolute ethanol and were put under ultrasonic vibrations for 60 min when cells were disintegrated by observing them microscopically. Then, the bound components to cells were released. The suspension was centrifuged at 15,000 rpm for 10 min and the obtained supernatant was filtered with 0.22-μm membranes for LC-MS analysis.

### 3.5. Mass Spectrometry

The fragment ions were analyzed by the UPLC/Q-TOF-MS method. The gradient elution system consisted of acetonitrile (solvent A) and water (solvent B). Separation was achieved using the following gradient procedures: 0.01–3 min, 10–30% A, 3–5 min, 30% A, 5–15 min, 30–45% A, 15–25 min, 45–95% A, 25–27 min, 95% A. The flow rate of mobile phase was set at 0.3 mL/min, and the column temperature was maintained at 40 °C. Ultrahigh pure helium (He) and high purity nitrogen (N_2_) were used as the collision gas and nebulizer, respectively. The optimized parameters in positive ion modes were as follows: fragmentor voltage, 100 V; skimmer voltage, 65 V; OCT 1 RF Vpp voltage, 750 V; capillary voltage, 5500 V; capillary temperature, 500 °C; atomizing pressure: 40 psi; dry nitrogen flow rate: 10 L/min; collision energy: 35 eV. Spectra were recorded in the range of *m*/*z* 50–1000 for full scan data.

### 3.6. Assay for LPS-Induced Cytokine Release of Macrophages

The effects of the three compounds—neoandrographolide, dehydroandrographolide, and β-sitosterol—on LPS-induced cytokine release of RAW264.7 cells were tested. The nitrite concentration in the culture medium was measured as an indicator of NO production according to the Griess reaction method described in a previous report [19]. The following: cells only, LPS alone (5 μg/mL), and LPS/drugs (1.25, 2.5, 5, 10, 20 μg/mL), were prepared as the control, model, and treatment groups, respectively. Six wells per group were used and 100 μL of the cells (2 × 10^5^ cells/mL) were added to each well. The plates were incubated for 24 h and 100 μL from the surface of each well was transferred into new plate, while 100 μL Griess reagent was added simultaneously. The new plate was then incubated for 10 min at room temperature and was measured by an ELISA reader at 540 nm. Standard calibration curves were prepared using sodium nitrite as the standard. NO inhibition (%) = [(model group)_NO_ − (treatment groups)_NO_]/(model group)_NO_ × 100%.

### 3.7. Statistical Analysis

All data were expressed as the mean ± standard deviations (SD) of six samples. The statistical analysis was performed with SPSS (SPSS statistical software package, version 18, SPSS Inc., Chicago, IL, USA). 

## 4. Conclusions

UPLC-Q/TOF-MS analysis is a rapid and effective technology for the recognition of complex chemical compounds from TCM. The application of UPLC/Q-TOF-MS greatly helps to improve the detecting efficiency and accuracy. Nine immunological active components in AH were simultaneously screened by macrophage biospecific extraction, and their immunological activity was tested through verification tests. Although there is no flavonoid isolate from AH, the flavonoids (compounds **8** and **9**) have been reported to interact with several enzymes, such as detoxification enzymes and plasma proteins [17,18]. So, as the configuration isomers, compounds **1**, **2**, and **5** may also exhibit similar interaction with proteins. Therefore, it is supposed that flavones from AH may also take part in the immunological active and play a bidirectional modulator in immune regulation. There may be a synergistic effect between flavonoids and diterpene lactones on the immune system. This study is the first reported work on immunological active components screening in AH. The potential immunological active of flavonoids from AH has not been reported previously. In conclusion, the application of cell-binding coupled with UPLC/Q-TOF-MS technology could be an efficient, rapid, and reliable method for screening complex mixtures from TCM.

## Figures and Tables

**Table 1:** Characteristics of potential bioactive compounds from AH by UPLC/Q-TOF-MS.

No.	t_R_ min	Molecular Formula	Molecular Ions *m*/*z*	Fragments *m*/*z*	Identification
1	8.5	C_24_H_26_O_11_	491.1540 [M + H]^+^ 513.1389 [M + Na]^+^	329.1012 [M + H − Glc]^+^ 314.0774 [M + H−Glc − CH_3_]^+^ Typical fragment 313.0683 Typical fragment 299.0544 Typical fragment 285.0743 Typical fragment 183.0250 Typical fragment 165.0162	Andrographidine E
2	8.9	C_25_H_28_O_12_	521.1649 [M + H]^+^	359.1119 [M + H − Glc]^+^ 344.0894 [M + H – Glc − CH_3_]^+^ 329.0658 [M + H – Glc − 2CH_3_]^+^ Typical fragment 315.0849 Typical fragment 285.0358 Typical fragment 183.0286 Typical fragment 165.0174	Andrographidine D
3	10.2	C_26_H_40_O_8_	481.2791 [M + H]^+^ 498.3056 [M + NH]^+^ 961.5517 [2M + H]^+^	319.2259 [M + H − Glc]^+^ Typical fragment 301.2155 Typical fragment 289.2155	Neoandrographolide
4	12.4	C_20_H_28_O_4_	333.2058 [M + H]^+^ 665.4045 [2M + H]^+^	315.1953 [M + H − H_2_O]^+^ 297.1846 [M + H − 2H_2_O]^+^ Typical fragment 285.1848 Typical fragment 257.1531	Dehydroandrographolide
5	13.0	C_19_H_18_O_6_	343.1170 [M + H]^+^	328.0942 [M + H − CH_3_]^+^ 313.0701 [M + H − 2CH_3_]^+^ 285.0751 [M + H − 2CH_3_-CO]^+^ Typical fragment 181.0128 Typical fragment 153.0175	5,7,2′,3′-tetramethoxyflavone
6	13.5	C_21_H_30_O_6_	379.2474 [M + H]^+^ 757.4880 [2M + H]^+^	361.2369 [M + H − H_2_O]^+^ 343.2291 [M + H − 2H_2_O]^+^ 315.1956 [M + H − 2H_2_O − CO]^+^ Typical fragment 297.1850 Typical fragment 285.1834 Typical fragment 257.1523 Typical fragment 175.1483 Typical fragment 133.1005	Unknown
7	18.8	C_29_H_50_O	415.2109 [M + H]^+^ 437.1930 [M + Na]^+^ 851.3967 [2M + Na]^+^	Typical fragment 135.0802 Typical fragment 119.0857	β-sitosterol
8	19.7	C_18_H_16_O_6_	329.1010 [M + H]^+^	314.0775 [M + H − CH_3_]^+^ Typical fragment 313.0697 299.0537 [M + H − 2CH_3_]^+^ Typical fragment 285.0743 Typical fragment 180.0038 Typical fragment 165.0167 Typical fragment 152.0091	5-hydroxy-7, 2′, 3′-trimethoxyflavone
9	20.0	C_19_H_18_O_7_	359.1116 [M + H]^+^	344.0890 [M + H − CH_3_]^+^ 329.0656 [M + H − 2CH_3_]^+^ Typical fragment 315.0864 Typical fragment 285.0387 Typical fragment 180.0047 Typical fragment 165.0177	5-hydroxy-7, 8,2′,3′-tetramethoxyflavone

**Table 2:** Effects of compounds **3**, **4**, and **7** on LPS-induced NO release of macrophages 1.

Groups	NO Level (μmol/L)			NO Inhibition (% of LPS)			NO IC_50_ (μg/mL)		
	3	4	7	3	4	7	3	4	7
Normal control (cells only)	0.52 ± 0.07	0.81 ± 0.11	0.58 ± 0.08	(-)	(-)	(-)			
LPS alone	13.46 ± 1.80 **	33.45 ± 0.76 **	17.90 ± 1.20 **	(-)	(-)	(-)			
LPS/drug (1.25 μg/mL)	11.68 ± 0.41	32.29 ± 1.22	17.59 ± 0.28	5.79 ± 0.80	5.79 ± 0.80	3.34 ± 0.28	9.03	18.18	**13.76**
LPS/drug (2.50 μg/mL)	11.01 ± 0.31	29.81 ± 2.11 ^##^	16.81 ± 0.81	13.71 ± 1.08	13.71 ± 1.08	9.64 ± 1.12			
LPS/drug (5.00 μg/mL)	9.28 ± 1.29 ^##^	29.06 ± 0.65 ^##^	12.49 ± 1.67 ^##^	13.13 ± 1.94	13.13 ± 1.94	38.42 ± 1.75			
LPS/drug (10.00 μg/mL)	5.79 ± 0.79 ^##^	24.07 ± 0.60 ^##^	9.74 ± 0.89 ^##^	28.04 ± 1.80	28.04 ± 1.80	45.59 ± 4.96			
LPS/drug (20.00 μg/mL)	1.95 ± 0.24 ^##^	15.70 ± 1.58 ^##^	3.56 ± 0.47 ^##^	53.06 ± 4.72	53.06 ± 4.72	80.09 ± 2.63			

** *p* < 0.01 vs. normal control group. ^##^ *p* < 0.01 vs. LPS alone group.

## Supplementary Materials

The following are available online, Figure S1: MS/MS spectra of andrographidine D (**2**), Figure S2: MS/MS spectra of dehydroandrographolide (**4**), Figure S3: MS/MS spectra of 5,7,2′,3′-tetramethoxyflavone (**5**), Figure S4: MS/MS spectra of compound **6**, Figure S5: MS/MS spectra of β-sitosterol (**7**), Figure S6: MS/MS spectra of 5-hydroxy-7, 2′, 3′-trimethoxyflavone (**8**), Figure S7: MS/MS spectra of 5-hydroxy-7, 8,2′,3′-tetramethoxyflavone (**9**).

## References

[1] Kumoro A.C., Hasan M. (2007). Supercritical carbon dioxide extraction of andrographolide from *Andrographis paniculata*: Effect of the solvent flow rate, pressure, and temperature. Chin. J. Chem. Eng..

[2] Wongkittipong R., Prat L., Damronglerd S., Gourdon C. (2000). Solid-liquid extraction of andrographolide from plants-experimental study, kinetic reaction and model. Sep. Purif. Technol..

[3] Hu X.G., Wen Y., Liu S.S., Luo J.B., Tan X.M., Li Z.H., Lu X.H., Long X.Y. (2015). Evaluation of the anaphylactoid potential of *Andrographis paniculata* extracts using the popliteal lymph node assay and P815 cell degranulation *in vitro*. J. Transl. Med..

[4] Xu Y., Chen A., Fry S., Barrow R.A., Marshall R.L., Mukkur T.K.S. (2007). Modulation of immune response in mice immunised with an inactivated Salmonella vaccine and gavaged with *Andrographis paniculata* extract or andrographolide. Int. Immunopharmacol..

[5] Sheeja K., Kuttan G. (2010). *Andrographis paniculata* down regulates proinflammatory cytokine production and augments cell mediated immune response in metastatic tumor-bearing mice. Asian Pac. J. Cancer P..

[6] Sunder J., Sujatha T., Rajas A., Kundu A. (2016). Immunomodulatory effect of Morinda citrifolia and *Andrographis paniculata* on expression of toll-like receptors in Nicobari fowl. Indian J. Anim. SCI..

[7] Wu T.S., Chern H.J., Damu A.G., Kuo P.C., Su C.R., Lee E.J., Teng C.M. (2008). Flavonoids and ent-labdane diterpenoids from *Andrographis paniculata* and their antiplatelet aggregatory and vasorelaxing effects. J. Asian Nat. Prod. Res..

[8] Santana M.T., Cercato L.M., Oliveira J.P. (2017). Medicinal Plants in the Treatment of Colitis: Evidence from Preclinical Studies. Planta Med..

[9] Zheng Z.G., Duan T.T., He B., Tang D., Jia X.B., Wang R.S., Zhu J.X., Xu Y.H., Zhu Q., Feng L. (2013). Macrophage biospecific extraction and HPLC–ESI-MS^n^ analysis for screening immunological active components in Smilacis Glabrae Rhizoma. J. Pharmaceut. Biomed..

[10] Chan K.M., Yue G.G.L., Li P., Wong E.C.E., Lee J.K.M., Kennelly E.J., Lau C.B.S. (2017). Screening and analysis of potential anti-tumor components from the stipe of *Ganoderma sinense* using high-performance liquid chromatography/time-of-flight mass spectrometry with multivariate statistical tool. J. Chromatogr. A..

[11] Yuan X.L., Zhang B., Wang Y.N., Ma J., Hou X.F. (2013). An online coupled breast cancer cell membrane chromatography with HPLC/MS for screening active compounds from *Fructus evodiae*. Anal. Methods..

[12] Dong Z.B., Zhang Y.H., Zhao B.J., Li C., Tian G., Niu B., Qi H., Feng L., Shao J.G. (2015). Screening for anti-inflammatory components from *Corydalis bungeana* Turcz. based on macrophage binding combined with HPLC. BMC Complement. Altern. Med..

[13] Li H.B., Yu Y., Wang Z.Z., Geng J.L., Dai Y., Xiao W., Yao X.S. (2015). Chemical profiling of Re-Du-Ning injection by ultra-performance liquid chromatography coupled with electrospray ionization tandem quadrupole time-of-flight mass spectrometry through the screening of diagnostic ions in MS^E^ mode. PLoS ONE.

[14] Song Y.X., Liu S.P., Jin Z., Qin J.F., Jiang Z.Y. (2013). Qualitative and quantitative analysis of *Andrographis paniculata* by rapid resolution liquid chromatography/time-of- flight mass spectrometry. Molecules.

[15] Arpini S., Fuzzati N., Giori A., Martino E., Mombelli G., Pagni L., Ramaschi G. (2008). HPLC-DAD-MS fingerprint of *Andrographis paniculata* (Burn. f.) Nees (Acanthaceae). Nat. Prod. Commun..

[16] Rao Y.K., Vimalamma G., Rao C.V., Tzeng Y.M. (2004). Flavonoids and andrographolides from *Andrographis paniculata*. Phytochemistry.

[17] Gokara M., Sudhamalla B., Amooru D.G., Subramanyam R. (2010). Molecular interaction studies of trimethoxy flavone with human serum albumin. PLoS ONE.

[18] Kotewong R., Duangkaew P., Srisook E., Sarapusit S., Rongnoparut P. (2014). Structure–function relationships of inhibition of mosquito cytochrome P450 enzymes by flavonoids of *Andrographis paniculata*. Parasitol. Res..

[19] Han S., Sung K.H., Yim D., Lee S., Lee C.K., Ha N.J., Kim K. (2002). The effect of linarin on LPS-induced cytokine production and nitric oxide inhibition in murine macrophages cell line RAW264.7. Arch. Pharm. Res..

