# Supplementary material for: Screening and Identification for Immunological Active Components from Andrographis Herba Using Macrophage Biospecific Extraction Coupled with UPLC/Q-TOF-MS
Source: Molecules. 2018 Apr 30;23(5):1047. doi: 10.3390/molecules23051047 (PMC6102597; doi:10.3390/molecules23051047)
Supplement: Supplementary file 1 [file molecules-23-01047-s001.pdf]

Supplementary Materials

Caption

Figure S1. MS/MS spectra of Andrographidine D (2)

Figure S2. MS/MS spectra of dehydroandrographolide (4)

Figure S3. MS/MS spectra of 5,7,2',3'-tetramethoxyflavone (5)

Figure S4: MS/MS spectra of compound 6

Figure S5: MS/MS spectra of β-sitosterol (7)

Figure S6: MS/MS spectra of 5-hydroxy-7, 2', 3'-trimethoxyflavone (8)

Figure S7: MS/MS spectra of 5-hydroxy-7, 8,2',3'-tetramethoxyflavone (9)

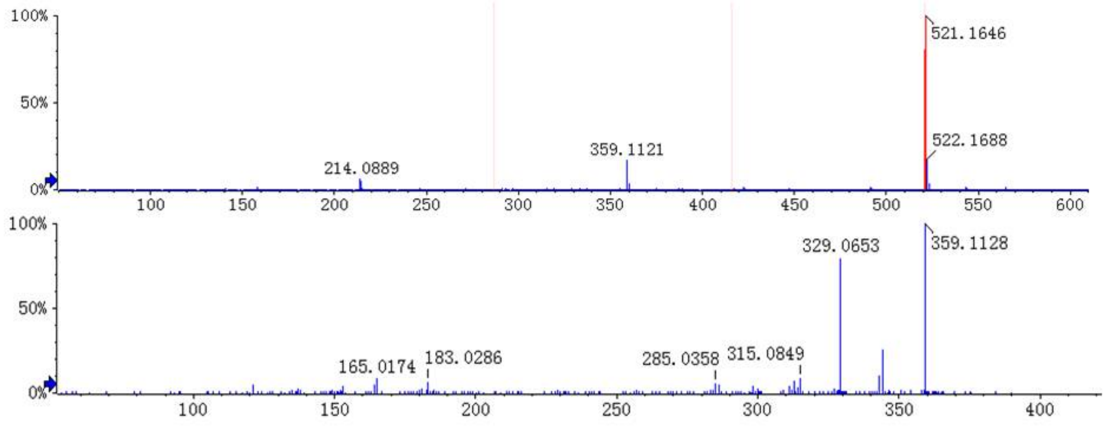

Figure S1. MS/MS spectra of Andrographidine D (2)

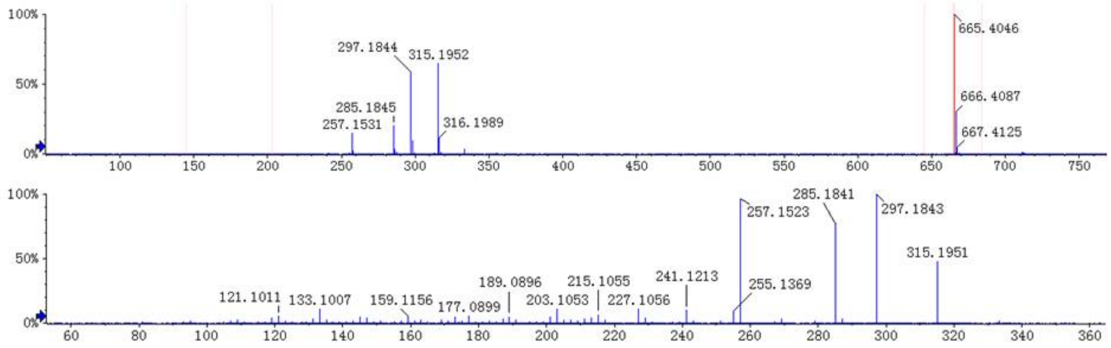

Figure S2. MS/MS spectra of dehydroandrographolide (4)

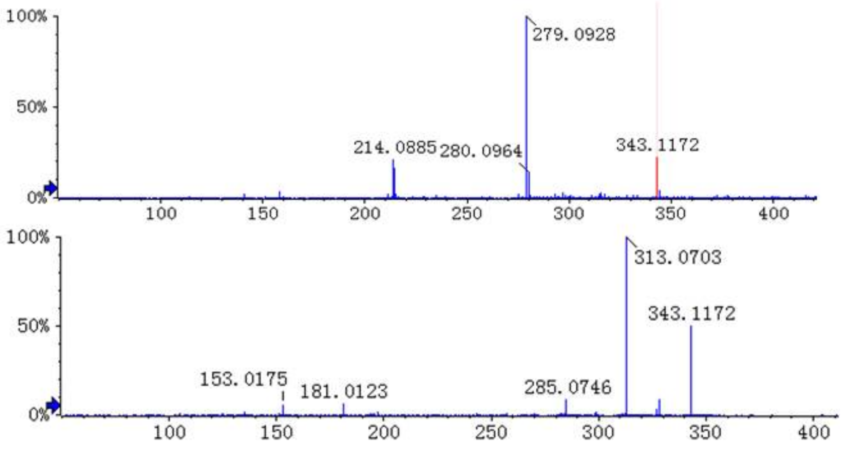

Figure S3. MS/MS spectra of 5,7,2’,3’-tetramethoxyflavone (5)

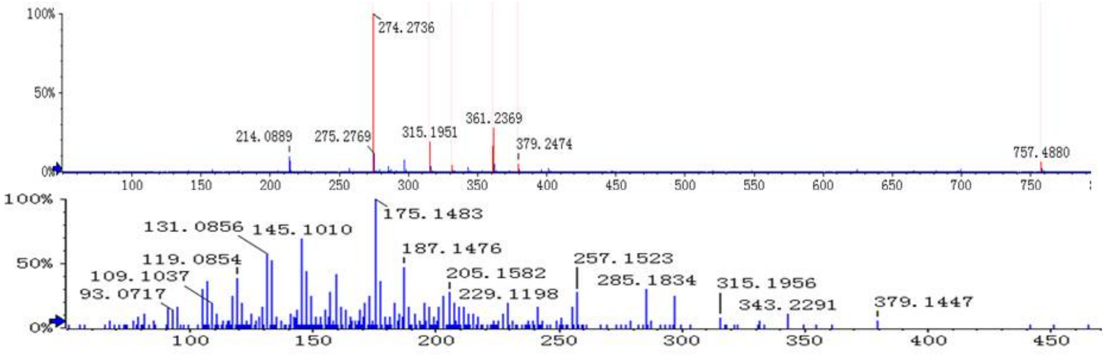

Figure S4: MS/MS spectra of compound 6

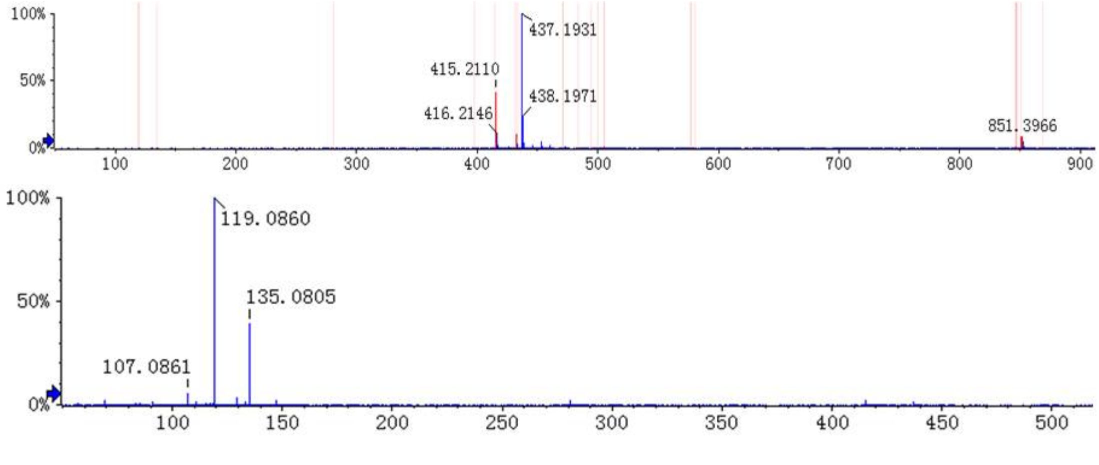

Figure S5: MS/MS spectra of β-sitosterol (7)

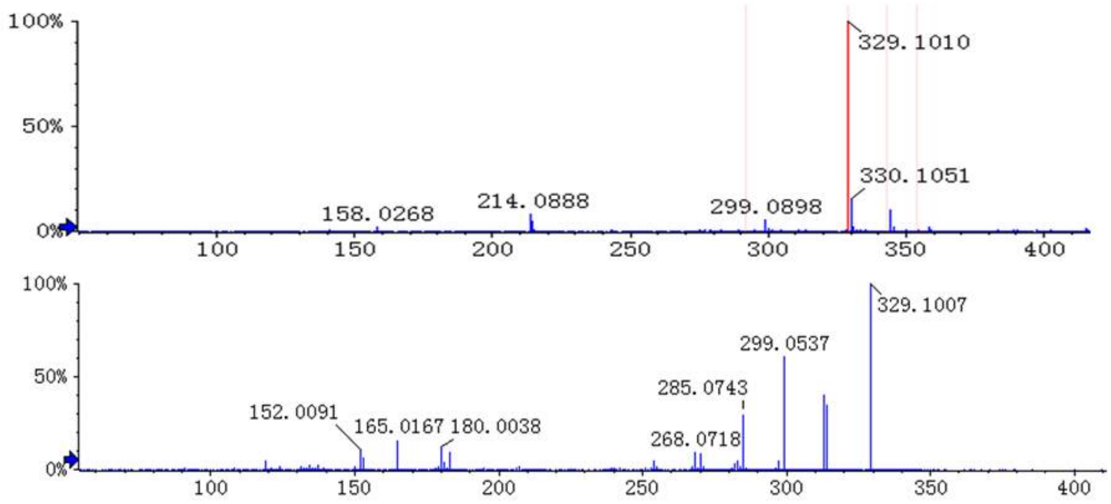

Figure S6: MS/MS spectra of 5-hydroxy-7, 2’, 3’-trimethoxyflavone (8)

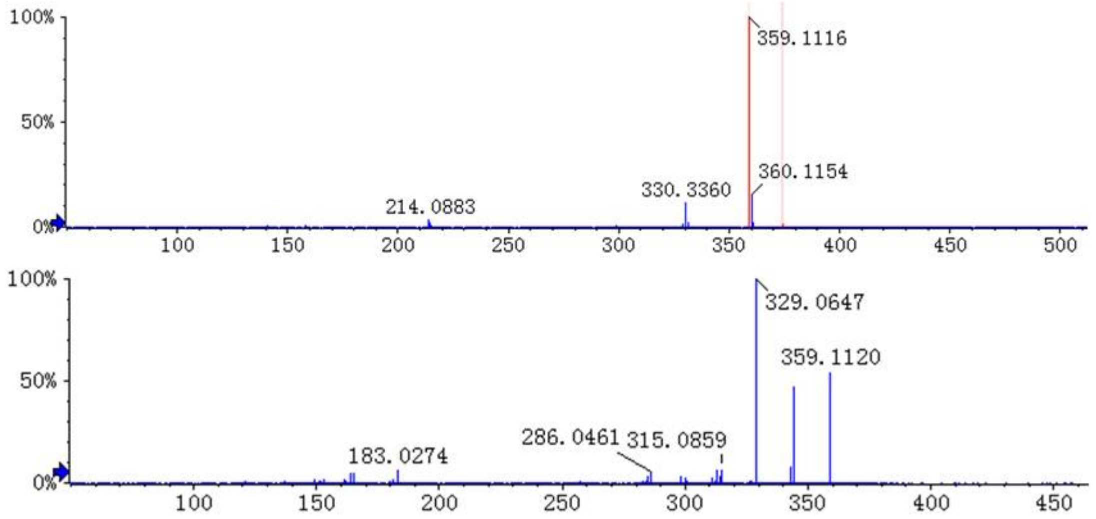

Figure S7: MS/MS spectra of 5-hydroxy-7, 8,2’,3’-tetramethoxyflavone (9)
